# The ANHEQ Evaluation Criteria: Introducing Reliable Rating Scales for Assessing Nordic Hamstring Exercise Quality

**DOI:** 10.1186/s40798-021-00383-x

**Published:** 2021-12-11

**Authors:** Tobias Alt, Marcus Schmidt

**Affiliations:** 1 Department of Biomechanics, Performance Analysis and Strength and Conditioning, Olympic Training and Testing Centre Westphalia, Dortmund, Germany; 2 Department for Sports and Sport Sciences, TU Dortmund University, Dortmund, Germany

**Keywords:** Evaluation tool, Eccentric resistance training, Hamstring strength, Execution quality, Adaptations

## Abstract

**Background:**

The Nordic Hamstring Exercise (NHE) is very popular for selective eccentric hamstring strengthening. However, NHE-related research is hindered by insufficient details about implementation and reporting. Available tools to assess study quality (e.g., PEDro or TESTEX scale) are too unspecific to account for the specific demands of NHE. Therefore, this study aimed to introduce two rating scales for Assessing Nordic Hamstring Exercise Quality (ANHEQ) of assessment and intervention studies.

**Methods:**

Eighteen graduated sports scientists, sports physiotherapists and elite coaches with scientific experience independently evaluated the quality of published NHE studies via ANHEQ scales, each comprising eight items and a maximal 13-point score. Inter-rater agreement was analyzed by using criterion-based reference values, while Krippendorff´s alpha determined inter-rater reliability. Systematic differences of the summated ANHEQ scores were determined using Friedman tests.

**Results:**

Inter-rater agreement was 87 ± 5% for NHE assessments and 88 ± 6% for interventions with single items ranging from 71 to 100%. Alpha values for inter-rater reliability ranged from fair (.250) to perfect (1.00) depending on the item. Total ANHEQ scores revealed coefficients of .829 (almost perfect) and .772 (substantial) without significant inter-rater differences (*p* = .292).

**Conclusions:**

The ANHEQ scales are suitable tools to rate NHE execution quality and data presentation. They facilitate a comprehensive review of NHE-related evidence and potentially improve the design and reporting of future NHE studies.

**Supplementary Information:**

The online version contains supplementary material available at 10.1186/s40798-021-00383-x.

## Keypoints

- Although the Nordic Hamstring Exercise (NHE) is very popular for selective eccentric hamstring strengthening, NHE-related research is hindered by insufficient details about implementation and reporting which cannot be assessed by recently available tools (e.g., Pedro or TESTEX scale).
- The present study introduced the ANHEQ scales which represent sensitive and reliable methods to rate NHE execution quality and data presentation and facilitate a comprehensive review of NHE-related evidence.
- Their application and interpretation are recommended for practitioners and scientists to evaluate the informative value of existing NHE-related evidence and to improve the design and reporting of future NHE studies as well as NHE execution in everyday training.

## Background

The Nordic Hamstring Exercise (NHE) is an effective resistance training exercise to improve hamstring strength, thigh muscle balance, as well as to mitigate hamstring strain and anterior cruciate ligament injury risk [1, 2]. First introduced in the late nineteenth century [3], NHE training has received growing research interest within the last 15 years because of its supramaximal eccentric intensity and because a similiar selective hamstring activation cannot be replicated by any other resistance exercise [4–6]. This supramaximal intensity is only realized if there is a break point (increased angular velocity), which for optimal exercise efficiency should be as close to the end of the ROM as possible. The break point is the knee flexion angle at which subjects are no longer able to maintain the required movement speed [7, 8]). However, most athletes demonstrate a premature ending of the controlled eccentric action of the NHE because of insufficient strength capacities [7–10]. This is a major limitation of current NHE studies to address because consistently high muscle activation in the injury-related extended knee angles (~ 30° to 0° knee flexion) represents an important target for prevention and rehabilitation [1, 6] to optimally mirror sport-specific demands [11]. Although NHE execution should always promote the ‘supramaximal’ stimulus characteristics, the ability to perform a full-ROM NHE mirrors the capacity to withstand high eccentric loads at extended knee angles which reduces the risk of muscle and knee injuries [1, 2, 5, 6]. Commonly, only ~ 50% of the NHE’s eccentric portion (range of motion to downward acceleration; ROM_DWA_) is executed in a controlled manner [7, 8, 10, 12]. Within the second half of the exercise, hamstring activation usually significantly declines and angular velocity continually increases [7, 8, 12]. These findings demonstrate that the physical demands of a single unassisted NHE exceed the physical capacities of most athletes [12].

Poor NHE execution technique (e.g., ROM_DWA_ less than 30°–45°, excessive hip flexion and lower back arch) and compliance might diminish or even prevent adaptations at long hamstring muscle length occurring at extended knee angles. Therefore, an assisted NHE execution is recommended to induce suitable adaptations and exercise-specific performance increases [9, 10, 13–16]. Apart from external assistance, an inclination of the shank reduces the eccentric load of the hamstrings and thus facilitates NHE execution [17].

Current NHE assessment and intervention studies often show a substantial lack of detail regarding implementation and reporting because information about execution modalities, testing procedures and data processing is imprecise or deficient. In the context of this study, we define assessments as studies which imply laboratory-based analyses of NHE performance and/or execution via kinematics, kinetics, electromyography and related procedures. In addition, NHE interventions are defined as studies which involve training regimens which focus on long-term adaptations, usually consisting of multi-week protocols, or prospective cohort studies.

In order to determine which assessment or intervention is effective, reproducible and trustworthy, it is mandatory to judge studies in terms of the standards of methodological and reporting quality. Therefore, a reliable rating scale assessing NHE quality is required to evaluate the informative and scientific value of existing NHE-related evidence and to improve future NHE studies. Existing tools which rate the quality of exercise training studies (e.g., PEDro, TESTEX) are too unspecific to account for the specific demands of proper and appropriate NHE execution [18, 19].

The aims of the present study were [1] to introduce rating scales for **A**ssessing **N**ordic **H**amstring **E**xercise **Q**uality (ANHEQ) of both NHE assessment and NHE intervention studies as well as (2) to determine their inter-rater agreement and inter-rater reliability. It is intended that both ANHEQ scales will support scientists as well as medical and performance practitioners to evaluate the quality of NHE-related research and to judge the existing evidence while conducting systematic reviews and meta-analyses. Furthermore, they should serve exercise science practitioners as specific guidelines for a targeted planning and implementation of acute and chronic NHE interventions.

## Methods

### Rating Scales and Criteria Selection

A series of five meetings between the members of the authorship group and four collaborative researchers were organized, during which quality and evaluation criteria were compiled for inclusion in the new rating scales. Items were selected to address the specific methodological problems and existing inaccuracies when assessing study quality of previously published NHE studies. Two separate rating scales for NHE assessment and intervention studies were subsequently developed:

- assessments: research studies which analyzed NHE performance and execution, usually performed under laboratory-based conditions and analyzed by biomechanical methods,
- interventions: research studies which implemented NHEs as a training exercise during multi-week interventions.

Both scales consist of eight items each assigned either scores of ‘2,’ ‘1’ or ‘0.’ The overall goal is to provide a graded and differentiated rating of study quality. Therefore, the most complex ANHEQ items 2, 5, 6 and 8 are scored by three-class scorings of ‘2,’ ‘1’ or ‘0.’ Items 3, 4 and 7 demand lower-complexity judgments and are therefore rated with ‘1’ or ‘0’ points. As Item 1 (rigid fixation of the heels) represents a characteristic which is crucially important for high-quality NHE execution [20], the upper score is weighted stronger, leading to scorings of ‘2’ or ‘0.’ In total, a maximum of 13 points is feasible in each ANHEQ scale. Most items were debated at several meetings until a consensus was reached and a draft protocol was circulated for comments. Three drafts were edited before a final version was reached. Once the draft was finalized, inter-rater agreement (IRA) as well as inter-rater reliability (IRR) was evaluated.

### Participants, Study Selection and Quality Assessment

Eighteen graduated sport scientists, sports physiotherapists and elite coaches (31 ± 4 years) with scientific experience of 7 ± 3 years (starting after their bachelor’s degree) volunteered to participate in the study. All observers independently evaluated the quality of eight published NHE studies using the ANHEQ scales, including four assessment [8, 17, 21, 22] and four intervention studies [20, 23–25]. The studies were selected from a list of 145 NHE studies because they demonstrated diverging NHE execution quality according to the ANHEQ scales. Since some of the selected studies analyzed multiple NHE assessments or interventions, the following conditions were rated: ‘NHD_30_’ [21] and ‘NHE variations A, D, F’ (referring to their Fig. 1) [17] for NHE assessments as well as ‘low volume intervention’ [23] and ‘progressive workload intervention’ [24] for NHE interventions. Each participant was provided with an information paper about the ANHEQ scales (Additional file 1), eight research papers and generic Excel spreadsheets on which to record their respective rating scores (Additional file 2).

**Fig. 1:**
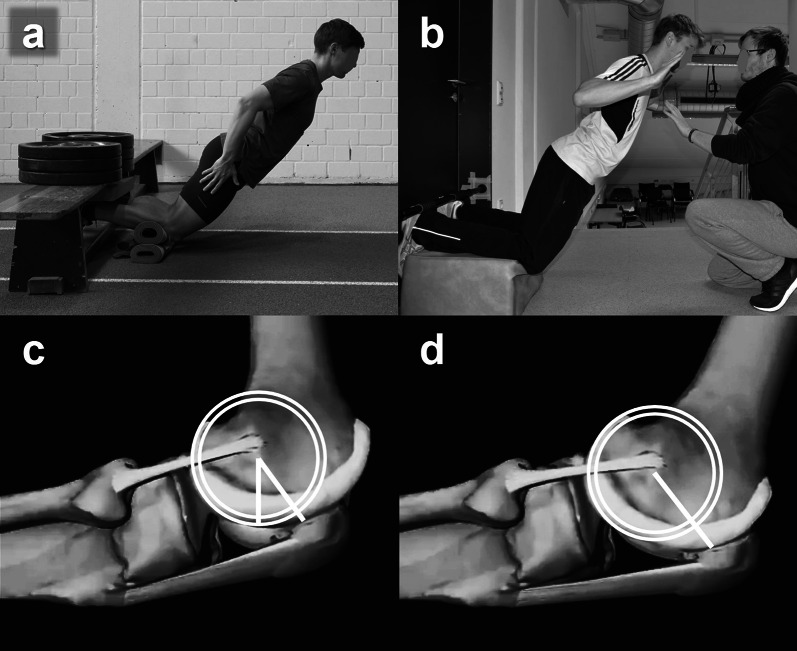
Exemplary NHE execution modalities with rigid resistance at the heels, but inappropriate (< 15 cm) (**a**) and appropriate kneeling height (≥ 15 cm) (**b**). A counter bearing of at least 140 kg is required to provide sufficient abutment for a 70 kg weighing athlete to perform an NHE until full knee extension (**a**). Assistance can be provided by a partner being located in front of the athlete and adjusting the pressure of his hands to the athlete’s shoulders according to the respective movement velocity (**b**). During flexion and extension, the rotational axis of the knee joint is not stationary and thus kneeling on a rigid floor throughout an NHE inhibits that the tibia head can smoothly roll underneath the patella (**c**, **d**)

### Inter-rater Agreement and Inter-rater Reliability

The distinction between IRA and IRR is a criterion-referenced interpretation of a rating scale. IRA is usually implemented in quantifying the informative value of evaluation tools, whereas IRR is frequently used in research studies which determine the consistency of observer ratings about the relative levels of performance [26].

As the level of quality in NHE studies is important, IRA was analyzed by using criterion-based reference values defined by the authors of this study. Each observer rating was compared with the reference value, and the percentage of absolute agreement was quantified as a measure for IRA. The percentage of absolute agreement is defined by calculating the number of times raters agree with the reference value divided by the total number of ratings. Thus, this measure varies between 0 and 100% and adds information about the rating accuracy to the IRR results [27].

IRR was assessed for each item available on both ANHEQ scales using the Krippendorff´s alpha coefficient. It counts pairs of categories that any number of raters have assigned to a single unit of analysis [28]. The coefficient is based on the following calculation:

$$\propto =1-\frac{{D}_{O}}{{D}_{\mathrm{e}}}$$

where *D*_0_ is the disagreement observed and *D*_e_ is the disagreement expected by chance. If the raters are in complete agreement, then $\propto$=1 [26]. In contrast to the commonly used Kappa statistics [18, 19, 29], Krippendorff’s alpha is applicable to samples with missing data, to any scale of measurement, and it takes sample size into account. As the first four items of both scales are identical, a shared analysis for NHE assessment and intervention studies was conducted. Accordingly, items 5 to 8 were analyzed separately for assessments and interventions. Secondary analyses for both scales were performed by assessing the IRR of their total ANHEQ scores. IRR was evaluated according to Landis and Koch [30]: > 0.80 ‘almost perfect’; 0.61–0.80 ‘substantial’; 0.41–0.60 ‘moderate’; 0.21–0.40 ‘fair’; 0.00–0.20 ‘slight’; and 0.00 ‘poor.’ Systematic differences of the total ANHEQ scale scores between the raters were determined by the nonparametric Friedman test. Data processing and statistical analyses were performed using R Software for statistical computing. The level of significance was set at *p* < 0.05.

## Results

### ANHEQ Criteria for NHE Assessment Studies

Table 1 summarizes the ANHEQ evaluation criteria for NHE assessments as well as the reference values of the four selected studies (A1 to A4). Additionally, an expanded version of the ANHEQ criteria can be found as a supplement of this paper (Additional file 1). It presents further details and precise descriptions how to apply the single items of the scoring system. Additional file 2 of this paper provides excel sheets to comfortably apply the ANHEQ scales. This file can be used by scientists, strength and conditioning coaches as well as physiotherapists to report the quality assessment of published research or to design their future NHE assessments and/or interventions. The following paragraphs provide specific and extensive information about the respective items of NHE assessments.

**Table 1:** Detailed information about the ANHEQ scale for NHE assessment studies and criterion-based reference values for studies A1 [8], A2 [21], A3 [17] and A4 [22]

Item	ANHEQ rating scales and explanation	Reference value			
		A1	A2	A3	A4
(1) Rigid fixation	*2 points*: fixed/rigid resistance at the heels *0 points*: partner assistance or not reported	0	2	2	2
(2) Knee position	*2 points*: feasible patellar glide (tuberositas tibiae placed on an edge, knees do not touch the floor) *1 point*: limited or partially feasible patellar glide (e.g., appropriately cushioned surface) *0 points*: patellar glide not feasible or not identifiable	0	0	1	1
(3) Kneeling height	*1 point*: shanks are placed at least 15 cm above the floor to enable full knee extension *0 points*: no/insufficient elevation or not identifiable	0	1	0	0
(4) Separate familiarization	*1 point*: a separate familiarization session was conducted to teach proper NHE technique *0 points*: instructions/ ‘familiarization’ at testing day or not reported	0	1	0	1
(5) Diagnostic tools	*2 points*: results of ≥ 2 diagnostic tools (kinematics, kinetics, electromyography) are presented *1 point*: results of 1 diagnostic tool (kinematics, kinetics, electromyography) are provided *0 points*: no diagnostic tool was applied or associated data are not reported	2	2	2	1
(6) Feedback of target movement speed	*2 points*: angle–time information is provided in real time to the participants by a monitor *1 point*: average cadence provided, e.g., by a metronome *0 points*: no feedback or not reported	0	2	0	0
(7) Consequences of impaired technique	*1 point*: defined consequences (e.g., repeated or excluded from analysis) *0 points*: unclear consequences or not reported	0	1	0	1
(8) Presentation of NHE performance variables	*2 points*: moment–angle or angle–time information (e.g., range of motion to downward acceleration) *1 point*: information about time under tension or range of motion *0 points*: no information available	2	1	2	0
**Total ANHEQ SCORE**		**4**	**10**	**7**	**6**

For A2 and A3, the ‘NHD_30_’ [21] and the ‘NHE variations A, D, F’ (referring to their Fig. 1) [17] were assessed, respectively

### Rigid Fixation (ANHEQ Item 1)

Appropriate execution of NHEs requires a rigid fixation of the heels, an important feature that has been addressed right from its first citation [3]. The fixed resistance ensures maximal force exertion across the greatest possible ROM. Predominantly, partner fixation does not suffice to provide a fixed abutment to perform an NHE across the full ROM (Fig. 1a) [20]. An inappropriate fixation of the heels causes a feeling of instability which will inevitably decrease muscle activation to avoid hurting oneself by an uncontrolled forward fall [6, 8, 17]. As a rigid fixation is essential to ensure valid and precise results, 2 points are awarded if the heels are placed against a rigid resistance (e.g., heel pads, ankle hooks, wall bars, doorway pull-up bars, step-bench, straps or any solid and rigid horizontal object) (Fig. 1b). Partner fixation, missing or imprecise information about the fixation gains 0 points.

### Knee Position (ANHEQ Item 2)

The knee position is a key component of NHE execution. If participants perform NHEs on a rigid surface, the pressure on the knees may cause uncomfortable feeling and pain and inhibits that the articular cartilage of the tibia head can smoothly roll underneath the patella and impedes a controlled execution in the middle portion of NHEs (~ 60° to 30° knee flexion). Consequently, the shanks should be placed on a cushioned, but not too soft surface which ends at the tibial tuberosity. Suitable positioning enables a physiological patella glide through the patellofemoral grove (Fig. 1c, d) [16, 20, 31–33]. Therefore, 2 points are awarded if the knee joints never touch the floor throughout an NHE across the full ROM. If the knee joints are not placed on an edge, but on an appropriately cushioned surface (e.g., foam pad, towel roll), which enables a limited or partially feasible patellar glide, 1 point is assigned. To receive 2 points or 1 point, NHE execution modalities must be clearly highlighted in a picture or a sketch and in ambiguous cases their characteristics should be explicitly mentioned in the methods. A sketch and/or a simple description like ‘cushioned/padded surface/board’ does not suffice to receive 1 point. An NHE execution on the floor or missing information deserves 0 points.

### Kneeling Height (ANHEQ Item 3)

Optimally, NHEs should be performed until nearly full knee extension while maintaining the highest possible activation of the hamstrings. This feature can be supported by an elevated kneeling height because every NHE is executed with a certain degree of hip flexion [9, 10, 17]. By achieving full knee extension, the head and chest will be below knee level and may hit the floor before completing full ROM (Fig. 1a). An elevated shank level of at least 15 cm is recommended, which matches the approximate height of two foam pads (Fig. 1b), a BOSU ball or related elevation [34, 35]. If participants perform NHEs with greater hip flexion (e.g., 20° to 40°), a larger kneeling height has to be chosen. Otherwise, the head and trunk will reach the floor prior to reaching full knee extension. One point is awarded if the shanks are placed at least 15 cm above the area which the chest and/or hands touch at full knee extension (provided that shanks are horizontally aligned). No or insufficient elevation (Fig. 1a) as well as missing information receives 0 points. Publications should enclose informative images, sketches, supplementary video material or should explicitly mention appropriate details to enable an assessment of the three aforementioned evaluation criteria.

### Separate Familiarization (ANHEQ Item 4)

Teaching proper NHE execution technique prior to the actual testing session ensures to get accurate, reliable and valid results from NHE studies. Therefore, a separate familiarization session is strongly recommended to improve inter alia motor imagery and neuromuscular activation [6, 17, 36]. This familiarization should include precise instructions, but above all a gradual accession process to proper exercise execution technique. Facilitations such as partner assistance or reduced range of motion should be used to convey the feeling for the movement (Fig. 1b). It is recommended to execute 2 sets of 3 repetitions across ~ 90° to 60° knee flexion followed by 3 sets of 3 assisted repetitions across the full ROM [9, 10]. A rest of ~ 6 s should be provided between repetitions and 5 min between sets. Due to potential delayed onset of muscular soreness, the familiarization session should be 3 to 7 days prior to the NHE assessment or the beginning of the NHE intervention period. Consequently, 1 point is awarded if it is clearly stated that at least a single familiarization session took place which included active NHE trials of the participants or that the participants were familiar with the specific procedures of the study. However, the latter approach is not recommended. It is advised to specify how familiarization took place and how many repetitions were performed. Descriptions like ‘warm-up/familiarization repetitions at testing day are performed,’ ‘participants were experienced/familiar with the NHE itself,’ ‘the exercise has been explained, demonstrated or shown during separate familiarization sessions’ or missing information deserves 0 points because these expressions do not stringently mean that the participants are familiar with the procedures, e.g., specific device, testing conditions of the NHE assessment.

### Diagnostic Tools (ANHEQ Item 5)

**Fig. 2:**
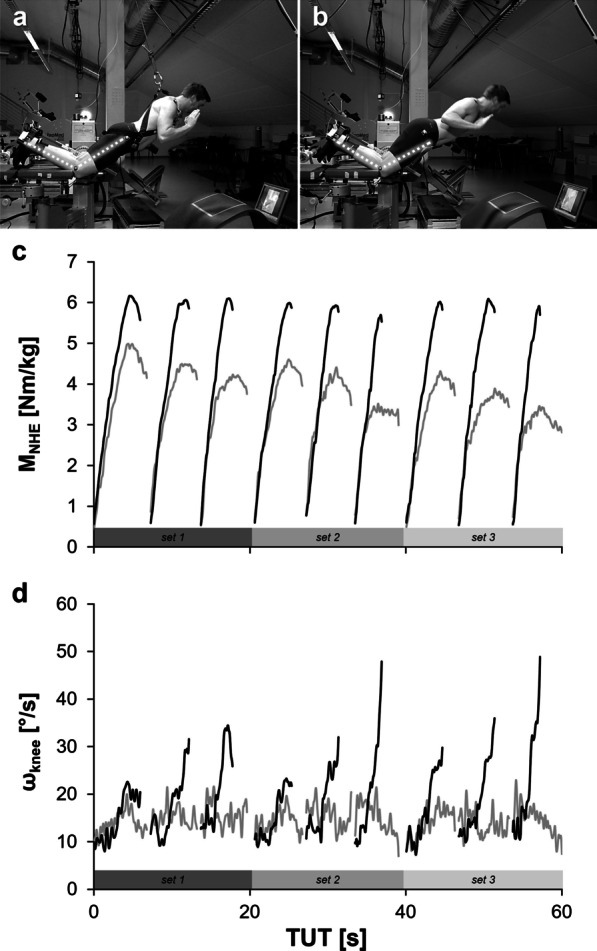
Representative illustrations of **a** assisted (grey lines) and **b** unassisted (black lines) NHE execution whose continuous angle–time information is monitored in real time on a monitor. Their exemplary **c** moment–time and **d** angular velocity–time histories are illustrated across 3 exemplary sets of 3 repetitions each. Reproduced with permission of Alt et al. [10]

Kinematic (motion capture or electro-goniometer), kinetic (measurement of force or moment) and electromyographic analyses provide manifold specific insights into NHE execution quality and are commonly investigated [6–10, 17]. The analysis of parameters like movement speed, time under tension, force generation until full knee extension or hip flexion angle characterizes NHE execution and contributes to evaluate how the presented values were generated [9–11, 21, 23, 37]. Points are assigned according to the number of implemented diagnostic tools (kinematics, kinetics, electromyography) whose data are presented in the text body of the manuscript, in a table or a figure: 2 points (≥ 2 diagnostic tools), 1 point (1 diagnostic tool) and 0 points (no diagnostic tool was applied or associated data were not reported).

### Feedback of Target Movement Speed (ANHEQ Item 6)

Standardized NHE test procedures should specify and supervise a constant target movement speed to get reliable results. Alterations of initial movement speed (until angle of downward acceleration) will impact force production due to altered hamstrings muscle–tendon unit stiffness [20, 21, 23, 38]. Therefore, it is recommended that a monitor provides an explicit position at each instance (angle–time information) in real time to the participants (Fig. 2a, b) [9, 10, 14, 21]. It is advised to present continuous information during the exercise where the participant's body should be situated at every single point in time. Additionally, if multiple repetitions are performed without feedback, fatigue will unavoidably lead to a continual increase in the average movement velocity during the controlled portion of NHEs (Fig. 2d). NHEs should be executed with slow and constant velocity. For optimal muscle–tendon adaptation, a single repetition should last 4 s to 6 s, while attaching special importance to the time under tension at knee flexion angles of 45° to 0° [38]. Two points are awarded if a figure, picture or sketch illustrates that continuous angle–time information is provided in real time to the participants (e.g., by a monitor) or if this information is given in the methods. Average cadence (e.g., provided by a metronome) deserves 1 point. While the use of a metronome is recommended, orally given cadence is tolerated as well. No feedback or missing information receives 0 points.

### Consequences of Impaired Technique (ANHEQ Item 7)

It should be defined which consequences occur if the aforementioned characteristics of high-quality NHE execution are not met. Assessment studies have to indicate if NHE repetitions were repeated or excluded from analysis if explicitly defined features were not fulfilled (e.g., inadequate hip flexion, lumbar lordosis, movement speed) [21, 22, 36]. Controlling execution quality is recommended to avoid an overload of the intervertebral discs of the lumbar spine [17]. One point is awarded if the consequences of impaired technique are defined (e.g., repeated or excluded from analysis). Optimally, a definition of parameters on how impaired technique is characterized (e.g., hip flexion of more than 30°, lower back arch, 20% deviation from target movement speed) is provided in the Methods section of the text body. Unclear consequences, or missing information about consequences of impaired technique is awarded with 0 points.

### Presentation of NHE Performance Variables (ANHEQ Item 8)

Even if execution modalities differ in terms of inter alia mean angular velocity, shank inclination or additional load, the same absolute force or moment values can be generated and vice versa. Moment–angle or angle–time information or related data provide important insights about how participants executed NHEs of assessment studies [8, 9, 12]. Therefore, it is recommended to present appropriate tables and/or figures (either in the main body or as supplementary material) illustrating representative or average data, which characterize NHE performance (Fig. 2c, d). The provided data contribute to comparing studies and judging the informative and scientific value of the presented results. Continuous graphs of moment–time or angle–time information deserve 2 points. Data of at least three averaged ROM epochs which are presented in the Results section are acceptable as well. If one-dimensional information about time under tension or range of motion is presented, 1 point is awarded. In the case of missing or inadequate information about NHE performance variables (e.g., a figure illustrating time as percentage of movement duration), 0 points are assigned.

### ANHEQ Criteria for NHE Intervention Studies

Table 2 summarizes the ANHEQ evaluation criteria for NHE interventions as well as the reference values of the four selected studies (I1 to I4). As previously presented for the assessment scale, an expanded version of Table 2 including detailed and more extensive descriptions of all criteria can be found as supplementary material (Additional file 1). As items 1 to 4 (Rigid Fixation, Knee Position, Kneeling Height and Separate Samiliarization) of NHE intervention studies are identical with NHE assessments, a duplication is avoided. The following paragraphs provide further specific and precise information about items 5 to 8 of NHE interventions.

**Table 2:** Detailed information about the ANHEQ scale for NHE intervention studies and criterion-based reference values for studies I1 [23], I2 [24], I3 [20] and I4 [25]

Item	ANHEQ rating scales and explanation	Reference value			
		I1	I2	I3	I4
(1) Rigid fixation	*2 points*: fixed/rigid resistance at the heels *0 points*: partner assistance or not reported	2	0	0	2
(2) Knee position	*2 points*: feasible patellar glide (tuberositas tibiae placed on an edge, knees do not touch the floor) *1 point*: limited or partially feasible patellar glide (appropriately cushioned surface) *0 points*: patellar glide not feasible or not identifiable	1	0	0	0
(3) Kneeling height	*1 point*: shanks are placed at least 15 cm above the floor to enable full knee extension *0 points*: no/insufficient elevation or not identifiable	0	0	0	0
(4) Separate familiarization	*1 point*: a separate familiarization session was conducted to teach proper NHE technique *0 points*: instructions/‘familiarization’ at testing day or not reported	1	0	0	0
(5) Progression and individualization of program variables	*2 points*: exercise intensity and/or volume progress and interindividual differences are assessed *1 point*: progression of exercise intensity and/or volume *0 points*: no progression or not reported	2	1	1	0
(6) Feedback of execution quality	*2 points:* visually (real-time feedback on a monitor) and audibly (e.g., by a coach or physiotherapist) *1 point*: visually (real-time feedback on a monitor) or audibly (e.g., by a coach or physiotherapist) *0 points*: no feedback or not reported	0	1	0	0
(7) Inter-set rest	*1 point*: adequate rest of ≥ 3 min (inter-repetition rest of ~ 6 s between eccentric NHEs) *0 points*: inadequate rest or not reported	0	0	0	0
(8) Compliance	*2 points*: participants performed ≥ 85% of NHEs repetitions *1 point*: participants performed 66–85% of NHEs repetitions *0 points*: participants performed < 66% of NHEs repetitions or not reported	2	2	2	0
**Total ANHEQ Score**		**8**	**4**	**3**	**2**

For I1 and I2, the ‘low volume intervention’ [23] and the ‘progressive workload intervention’ [24] have been assessed, respectively

### Progression and Individualization of Program Variables (ANHEQ Item 5)

As athletes improve their physical work capacity throughout regular resistance training, the relative exercise intensity as well as the exercise volume should be individually adapted over time. Therefore, continuous assessments of work capacity should be undertaken during the intervention and the exercise load should be adjusted accordingly [19]. Therefore, it is recommended to provide information about individual training loads and volumes (optimally as averaged or summed time under tension, moment, force and/or impulse over time) in the main body or as supplementary material [23]. Optimal muscle–tendon training programs should include high intensity by keeping the exercise volume rather low (≤ 5 repetitions per set) [9, 10, 34, 38, 39]. Instead of increasing the number of repetitions, the exercise volume should be adapted via total impulse and/or time under tension on the individual capacities of the participants (Fig. 2c). Providing external assistance [9, 10, 13–16] and/or reducing the initial knee flexion angles (e.g., by increased shank inclination) [17] are appropriate tools for participants who want to focus on force production at extended knee angles and/or are not strong enough to complete NHEs across the full range of motion.

Two points are awarded for completing and reporting periodic adjustments of exercise intensity and volume according to individual capacities. Contradicting the intended purpose of high-intensity NHE training, individual variations (additional weights or higher initial movement speed) at relatively high repetition numbers (> 6) are not awarded with 2 points. If exercise intensity and/or volume progresses without individual adjustments or insights into interindividual differences, 1 point is assigned for this item. A simple statement that stronger athletes should perform more repetitions per set than beginners (e.g., FIFA 11+) is not awarded with 1 point. Intervention studies, which did neither adapt nor report a progression of program variables, receive 0 points.

### Feedback of Execution Quality (ANHEQ Item 6)

As mentioned in the ANHEQ item 6 for NHE assessments (Feedback of Target Movement Speed) high-quality NHEs are performed with constant slow movement speed until full knee extension (20° to 0° knee flexion) while maintaining a defined hip flexion (e.g., 20°) and a physiological lordosis of the lumbar spine [15, 17]. Feedback of these NHE execution parameters is crucial to ensure appropriate execution quality and should therefore be continuously provided during training interventions [9]. Two points are awarded if it is explicitly stated that real-time feedback was given visually and audibly. Furthermore, additional information should be presented how the feedback was provided (e.g., on a monitor, by a coach, physiotherapist or a metronome including details about the tools, specific aspects and/or purpose of feedback) (Fig. 2a, b). One point is assigned if the feedback is only presented in one way, either visually or audibly (e.g., if the authors indicate that feedback was provided by a coach or physiotherapist). A short statement that the NHE training was supervised without any details about extent or content of feedback does not suffice to receive 1 point. In the case of missing feedback or no information about given feedback, the intervention study gets 0 points.

### Inter-set Rest (ANHEQ Item 7)

The amount of rest between sets and exercises significantly affects the metabolic and the hormonal responses to an acute bout of resistance exercise [40]. Rest period length significantly influences muscular strength and accumulating fatigue. Therefore, if the resistance exercise program is designed for power, 5 min to 8 min is appropriate, whereas 3 min to 5 min is required for maximal strength [41, 42]. As a single unassisted NHE induces high intensity and internal load [10], an inter-set rest of at least 3 min is recommended. Due to the passive return into the starting position, eccentric-only NHE training usually implies an inter-repetition rest of ~ 6 s between repetitions. If additional inter-repetition rest is granted to avoid excessive fatigue, this should be mentioned. Intervention studies which provide an inter-set rest of ≥ 3 min deserve 1 point for this item. It is advised to provide detailed information about inter-set and inter-repetition rest periods. If an inter-repetition rest of > 6 s was guaranteed, an inter-set rest period of ≥ 2 min is also awarded with 1 point. Inadequate or not reported rest periods receive 0 points.

### Compliance (ANHEQ Item 8)

Compliance to total volume and duration of NHE training interventions is inevitable to ensure intended adaptations and performance enhancement over time [1, 33, 43]. Due to extended intervention periods typical for NHE interventions, the proportion of withdrawals is often remarkable high. Quite often more than 15% of participants will withdraw from an exercise training study during the stipulated study period [19]. Moreover, exercise attendance is less than 85% in some of the participants who do not withdraw from the study [19]. For the purposes of ANHEQ scales, compliance is defined as the percentage of target repetitions completed by each individual who was included in the analysis. Participants, who did not pass the complete intervention period and were excluded from data analysis, should be mentioned separately while specifying the reasons for withdrawal. Studies with intervention compliances of at least 85% deserve 2 points. One point is awarded if compliance is less than 85% but ≥ 66%. It is recommended that in both cases, adverse events are reported, which are directly connected to the intervention program (e.g., injuries or DOMS). If intervention attendance is less than 66% or no information about compliance is given, 0 points will be awarded. In the case of interventions which include the NHE as obligatory or optional part of a multi-exercise regimen, the NHE-related compliance should be presented. If only the compliance with the entire intervention program is reported, 0 points are awarded as well.

### Overall Rating Guidelines and Interpretation

For all presented ANHEQ items, it is intended that in case of doubts about the awarding of 2, 1 or 0 points, always the inferior grading should be applied. Publications should enclose informative images, sketches, supplementary video material or should explicitly mention appropriate details to enable an accurate rating according to the ANHEQ items. If a reference is made to an existing study, detailed descriptions should be added to receive the grading of the referenced study. Otherwise 0 points are awarded. To judge the overall quality of NHE assessments and interventions, the total ANHEQ scores are allocated to grades according to the American College Grading System: 12/13 points ‘excellent’; 11/10 points ‘very good’; 9/8 points ‘good’; 7/6 points ‘average’; 5/4 points ‘below average’; 3/2 points ‘poor’; 1/0 points ‘failure’. The excel file which is attached as Additional file 2 can be easily used to apply the ANHEQ criteria and judge published research papers. It provides an automated evaluation according to the grading system.

### Inter-Rater Agreement and Inter-Rater Reliability

With regard to the items for NHE assessment studies, the IRA to the criterion-based reference values was at least 71% rising to perfect agreement (100%). Concerning the NHE intervention scale, minimal agreement of single items was 75%. The lowest IRA for a single rating item became apparent for item 2 of study A2 (44%) and for item 6 of study I1 (56%) (Table 3), respectively. The overall agreement of the 18 raters laid between 87 ± 5% (78–97%) for NHE assessments and 88 ± 6% (75–100%) for NHE interventions (Table 4).

**Table 3:** Item-specific information about inter-rater agreement displayed as percentage of absolute agreement [%] to the criterion-based reference values of the selected NHE assessment and intervention studies

ANHEQ Item	NHE Assessment Studies					ANHEQ Item	NHE Intervention Studies				
	A1 [8]	A2 [21]	A3 [17]	A4 [22]	Total		I1 [23]	I2 [24]	I3 [20]	I4 [25]	Total
(1) Rigid fixation	100	100	100	100	**100**	(1) Rigid fixation	100	100	100	83	**96**
(2) Knee position	100	44	50	89	**71**	(2) Knee position	83	100	72	89	**86**
(3) Kneeling height	100	50	50	89	**72**	(3) Kneeling height	94	100	100	89	**96**
(4) Separate familiarization	100	100	89	89	**94**	(4) Separate familiarization	100	83	50	100	**83**
(5) Diagnostic tools	100	94	100	72	**92**	(5) Progression and individualization of program variables	67	72	61	100	**75**
(6) Feedback of target movement speed	89	89	94	100	**93**	(6) Feedback of execution quality	56	67	100	83	**76**
(7) Consequences of impaired technique	100	83	100	100	**96**	(7) Inter-set rest	100	100	100	100	**100**
(8) Presentation of NHE performance variables	100	50	56	100	**76**	(8) Compliance	89	94	100	89	**93**
**Mean**	**99**	**76**	**80**	**92**	**87**	**Mean**	**86**	**90**	**85**	**92**	**88**
**SD**	**4**	**23**	**22**	**9**	**11**	**SD**	**16**	**13**	**20**	**7**	**9**

**Table 4:** Rater-specific information about inter-rater agreement displayed as percentage of absolute agreement [%] to the criterion-based reference values of the selected NHE assessment and intervention studies

Rater	NHE Assessment Studies								Total	NHE Intervention Studies								Total
	Item 1	Item 2	Item 3	Item 4	Item 5	Item 6	Item 7	Item 8		Item 1	Item 2	Item 3	Item 4	Item 5	Item 6	Item 7	Item 8	
#1	100	75	75	100	100	100	100	100	**94**	100	75	100	50	50	75	100	100	**81**
#2	100	75	75	100	75	75	100	100	**88**	100	75	75	75	75	100	100	100	**88**
#3	100	100	75	100	50	100	100	75	**88**	100	75	100	75	100	75	100	100	**91**
#4	100	100	75	100	100	100	75	75	**91**	75	100	100	100	100	100	100	75	**94**
#5	100	50	50	100	100	100	100	75	**84**	100	100	100	100	75	75	100	100	**94**
#6	100	50	75	100	75	100	100	50	**81**	100	100	100	100	100	100	100	100	**100**
#7	100	50	50	75	100	100	100	75	**81**	100	100	100	100	75	75	100	100	**94**
#8	100	75	50	100	100	100	100	50	**84**	100	75	100	75	75	75	100	100	**88**
#9	100	75	100	100	75	50	100	50	**81**	100	100	100	75	50	75	100	100	**88**
#10	100	75	75	100	100	100	100	100	**94**	100	100	100	75	75	100	100	50	**88**
#11	100	50	100	75	100	75	100	100	**88**	75	50	100	100	75	50	100	100	**81**
#12	100	50	50	100	100	100	75	75	**81**	100	75	100	75	75	100	100	75	**88**
#13	100	100	50	100	75	100	100	50	**84**	100	100	100	75	75	50	100	100	**88**
#14	100	75	100	100	100	100	100	75	**94**	100	75	100	75	100	75	100	100	**91**
#15	100	75	75	100	100	100	100	75	**91**	75	75	100	75	50	50	100	75	**75**
#16	100	50	50	75	100	75	100	75	**78**	100	100	100	100	75	100	100	100	**97**
#17	100	75	100	100	100	100	100	100	**97**	100	75	100	100	50	50	100	100	**84**
#18	100	75	75	75	100	100	75	75	**84**	100	100	50	75	75	50	100	100	**81**
**Mean**	**100**	**71**	**72**	**94**	**92**	**93**	**96**	**76**	**87**	**96**	**86**	**96**	**83**	**75**	**76**	**100**	**93**	**88**
**SD**	**0**	**18**	**19**	**11**	**15**	**14**	**10**	**18**	**5**	**10**	**15**	**13**	**15**	**17**	**20**	**0**	**14**	**6**

As presented in Table 5, inter-rater reliability ($\propto$) ranged from 0.250 (fair) to 1.00 (perfect). The fair coefficient occurred in the items ‘Kneeling Height’ (both scales) and ‘Feedback of Execution Quality’ (intervention scale). Ratings for ‘Knee Position’ resulted in moderate (0.421) reliability. Item 7 ‘Inter-set Rest’ of the intervention scale showed perfect reliability. Therefore, confidence interval and p value could not be calculated for this item. Five of the twelve categories (42%) reached substantial agreements (≥ 0.61), while three (25%) showed almost perfect agreement to the respective reference values. Concerning the total ANHEQ scores, $\propto$ coefficients of 0.829 (almost perfect) and 0.772 (substantial) were reached for the assessment and intervention scale, respectively. The total ANHEQ scores revealed no significant differences between the observers (Friedman chi-squared = 19.663, *df* = 17, *p* = 0.292).

**Table 5:** Item-specific information about inter-rater reliability of all ANHEQ scale items across the 18 raters

ANHEQ Item	Applicable to	Points	*α* (± SE)	Confidence interval	*p* value
1) Rigid fixation	Assessment and Intervention	2 or 0	0.924 (0.060)	(0.783, 1.000)	0.000
2) Knee position	Assessment and Intervention	2, 1 or 0	0.421 (0.089)	(0.211, 0.631)	0.002
3) Kneeling height	Assessment and Intervention	1 or 0	0.266 (0.041)	(0.17, 0.361)	0.000
4) Separate familiarization	Assessment and Intervention	1 or 0	0.690 (0.104)	(0.443, 0.936)	0.000
5) Execution quality assessment	Assessment	2, 1 or 0	0.718 (0.096)	(0.412, 1.000)	0.005
Progression and individualization of program variables	Intervention	2, 1 or 0	0.604 (0.183)	(0.022, 1.000)	0.046
6) Feedback of target movement speed	Assessment	2, 1 or 0	0.878 (0.057)	(0.698, 1.000)	0.001
Feedback of execution quality	Intervention	2, 1 or 0	0.250 (0.126)	(-0.151, 0.650)	0.142
7) Consequences of impaired technique	Assessment	1 or 0	0.854 (0.126)	(0.454, 1.000)	0.006
Inter-set rest	Intervention	1 or 0	1.000	–	–
8) Presentation of NHE performance variables	Assessment	2, 1 or 0	0.739 (0.200)	(0.104, 1.000)	0.034
Compliance	Intervention	2, 1 or 0	0.737 (0.069)	(0.517, 0.957)	0.002
**Total**	Assessment	**max. 13**	**0.829 (0.079)**	**(0.577, 1.000)**	**0.002**
	Intervention	**max. 13**	**0.772 (0.084)**	**(0.503, 1.000)**	**0.003**

## Discussion

The Nordic Hamstring Exercise is a key component of eccentric hamstring strengthening [1–3]. Although frequently implemented in research and training [4–6], NHE execution quality is often neither precisely reported nor purposive to induce best possible adaptations. Therefore, the aims of the present study were [1] to introduce rating scales for **A**ssessing **N**ordic **H**amstring **E**xercise **Q**uality (ANHEQ) of both NHE assessments and NHE interventions as well as [2] to determine their inter-rater agreement and inter-rater reliability. By the use of these scales, scientists and practitioners can rate NHE execution quality of published research and consequently improve the design and reporting of future NHE studies and, above all, NHE execution in everyday testing and training.

### IRA and IRR of ANHEQ Scales

The inter-rater agreement to the criterion-based reference values was generally high for NHE assessments (87 ± 5%) and interventions (88 ± 6%) (Table 4). It became apparent that inconsistent ratings predominantly occurred in single studies and items (e.g., Item 2 and 3 of study A2 and A3; Item 6 of study I1 and I2) (Table 3). Depending on the item, inter-rater reliability of the different ANHEQ items ranged from fair (0.250) to perfect (1.00) (Table 5). The presented values are comparable with previous studies about the development and validation of tools for the assessment of study quality and reporting in exercise studies [18, 19]. Consequently, observers can achieve appropriate levels of agreement and reliability reflecting the clarity of each ANHEQ rating scale item. The fair coefficients of IRR for the items ‘Kneeling Height’ and ‘Feedback of Execution Quality’ as well as low percentages of agreement to criterion-based reference values can be traced back to imprecise reporting (e.g., unclear or misleading descriptions, insufficient or missing figures of NHE execution) or defficient information provided by the selected publications. Total ANHEQ scores revealed $\propto$ coefficients of 0.829 (almost perfect) and 0.772 (substantial), emphasizing that the overall ratings of NHE study quality are reliable for both assessments and interventions (Table 5). In general, the presented ANHEQ scales were able to consistently assess quality of NHE assessment and intervention studies because no significant differences between observers (Friedman chi-squared = 19.663, *df* = 17, *p* = 0.292) became apparent. The present study included ratings of a heterogeneous group of graduated sports scientists, sports physiotherapists and elite coaches. Systematic errors due to subjective appraisals of provided cushioning (ANHEQ Item 2: ‘﻿Knee Position﻿’) and ‘﻿Kneeling Height﻿’ (ANHEQ Item 3) might have led to inconsistent ratings. The rating order of the eight studies was not randomized (assessment prior to intervention studies) so that a certain degree of familiarization or rating adjustment might have occurred. However, the ANHEQ rating scales with their newly introduced items address common shortcomings in study design, quality and reporting of NHE studies.

### Perspectives

Future NHE studies should apply the ANHEQ criteria for appropriate planning, conducting and reporting. They ought to reveal if an assisted (e.g., by means of an elastic band) or unassisted execution is more effective in promoting eccentric hamstring strength and musculo-tendinous adaptations [9, 10, 13–16﻿]. Generally, sufficiently strong participants (ROM_DWA_ > 45°) have not been investigated yet to prove which NHE execution modalities will lead to the best adaptations:

- unassisted vs. assisted,
- neutral vs. flexed hip,
- unloaded vs. loaded,
- slow vs. fast velocity,
- ﻿bilateral vs. unilateral,
- constant velocity vs. decelerated execution.

Future NHE assessments should more frequently investigate common performance variables such as force, time under tension and impulse to mirror the execution quality of their implemented trials. These data are readily available from specific devices which are feasible in both applied and laboratory settings. Prospective NHE interventions should determine individual relationships between training loads and performance as well as related physical parameters to recognize responders and non-responders [35]. Finally, the transfer of NHE-induced improved hamstring strength to sport-specific tasks such as sprinting is of major interest [14]. But most importantly, future NHE assessments as well as interventions should be conducted according to the ANHEQ criteria to allow better replication and understanding of study quality.

### Practical Recommendations

The ultimate goal of NHE assessments and interventions should be to promote optimal exercise execution including a permanently high muscle activation across the entire ROM to maximally stress the hamstrings muscle–tendon unit, especially at extended knee angles (~ 30° to 0° knee flexion) [8, 9, 23]. Load management of unassisted NHEs is a dilemma between high intensity and the specific muscle activation patterns at extended knee angles. Certainly, practitioners and scientists should promote the ‘supramaximal’ stimulus characteristics of the NHE. However, special emphasis should always be directed toward the activation and time under tension at injury-relevant longer muscle lengths [1, 2, 5, 6]. Due to the well-demonstrated positive effects of eccentric movements, the high physical demands and the fatiguing character of NHEs, it is advised to perform just the descent part of the NHE in an active fashion [9, 10, 12, 14, 24].

NHE assessments and interventions on specific devices [6, 17, 21, 22﻿] or isometric dynamometers [9, 10, 14] (Fig. 2a, b) are recommended to quantify exercise determinants, performance parameters and exercise quality. If no specific device is available, wall bars, doorway pull-up bars or any other solid and rigid horizontal object (Fig. 1a, b) can serve to provide adequate counter bearing for the heels. Selecting a knee position on a cushioned surface, which ends at the tibial tuberosity, is more physiological (Fig. 1c, d; ANHEQ Item 2: ‘﻿Knee Position﻿’﻿).

Assisted NHE execution—e.g., provided by a partner being located in front of the athlete and adjusting the pressure of his hands to the athlete’s shoulders according to the respective movement velocity (Fig. 1b) or by means of an elastic band [13, 15, 16]—is recommended to acquire proper exercise quality, to reduce the fear of uncontrolled falling and to increase the actively controlled range of motion, especially within early training stages of inexperienced athletes or patients [9, 10, 13, 14].

Due to accumulating inter-set fatigue during unassisted NHEs which highly varies between participants, it might be more reasonable to use muscular failure—associated with a large increase in angular velocity at the end of a repetition (Fig. 2d)—as completion criterion of a set rather than a prescribed repetition number [10, 44]. This might contribute to more individualized NHE training regimen respecting actual daily performance.

## Conclusions

The present study introduced rating scales for NHE assessment and intervention studies to improve the reporting and execution quality of future research. **A**ssessing **N**ordic **H**amstring **E**xercise **Q**uality via ANHEQ scales represents a sensitive and reliable method to rate NHE study quality and data presentation. Their application and interpretation are recommended for scientists as well as medical and performance practitioners to evaluate the informative value of existing NHE-related evidence and to improve the design and reporting of future NHE studies as well as NHE execution in everyday training.

## Supplementary Information

**Additional file 1.** Expanded description of the ANHEQ criteria.

**Additional file 2.** Generic ANHEQ Rating Spreadsheets.

## Data Availability

The data that support the finding of this study are not publicly available due to privacy or ethical restrictions but are available from the corresponding author on reasonable request.

## Abbreviations

ANHEQ: Assessing Nordic Hamstring Exercise Quality
DOMS: Delayed onset of muscle soreness
IRA: Inter-rater agreement
IRR: Inter-rater reliability
NHE: Nordic hamstring exercise
ROM: Range of motion
ROM_DWA_: Range of motion to downward acceleration
