# Supplementary material for: The ANHEQ Evaluation Criteria: Introducing Reliable Rating Scales for Assessing Nordic Hamstring Exercise Quality
Source: Sports Med Open. 2021 Dec 11;7:91. doi: 10.1186/s40798-021-00383-x (PMC8665968; doi:10.1186/s40798-021-00383-x)
Supplement: Supplementary file 1 — Additional file 1. Expanded description of the ANHEQ criteria. [file 40798_2021_383_MOESM1_ESM.docx]

**Tab. S1**. Detailed explanations of all ANHEQ Items

|  | **ANHEQ Item** | **applicable to** | **ANHEQ rating scale & explanation** |
| --- | --- | --- | --- |
| **ITEM 1** | Rigid fixation | Assessment and Intervention | 2 points: fixed/rigid resistance at the heels   - must be clearly visible in a picture or sketch or must be explicitly mentioned in the methods - e.g., bars, wall bars, doorway pull-up bars, step-bench, straps or any solid and rigid horizontal object which may move in antero-posterior direction, but should limit displacement in vertical direction   0 points: partner assistance or not reported |
| **ITEM 2** | Knee position | Assessment and Intervention | 2 points: feasible patellar glide (tuberositas tibiae placed on an edge, knee do not touch the floor)   - must be clearly visible in a picture or sketch or/and (in ambiguous cases) must be explicitly mentioned in the methods   1 point: limited or partially feasible patellar glide (e.g., appropriately cushioned surface)   - must be visible in a picture (e.g., minimal cushion provided by towel roll, NordBord or related cushion) or sort of cushion/surface must be mentioned in the methods - a sketch and/or a simple description like "cushioned/padded surface/board” does not suffice to receive 1 point   0 points: patellar glide not feasible or cushion's degree not identifiable   - a short reference to an existing study/methodology without any specific details about cushion |
| **ITEM 3** | Kneeling height | Assessment and Intervention | 1 point: shanks are placed at least 15 cm above the floor to enable full knee extension   - must be clearly visible in a picture or sketch or/and (in ambiguous cases) must be explicitly mentioned in the methods - e.g., two foam pads, Bosu-ball or related elevation   0 points: no/insufficient elevation or not identifiable   - a short reference to an existing study/methodology without any appropriate details about kneeling height |
| **ITEM 4** | Separate familiarization | Assessment and Intervention | 1 point: a separate and active familiarization session was conducted to teach proper NHE technique   - must be clearly stated that at least a single familiarization session took place (>48h prior to test session) which included active NHE trials of the participants or must be clearly stated that the participants were familiar with the procedures (HOWEVER this approach is not recommended!) - It is recommended to specify how familiarization took place and how and how many repetitions were performed   0 points: instructions/‘familiarization‘ at testing day or not reported   - warm-up/familiarization repetitions at testing day are performed (this procedure does not induce improved motor imagery and neuromuscular activation) - simply mentioning that participants were experienced/familiar with the NHE itself (a familiarization to the procedures, e.g., specific device, testing conditions of the the NHE intervention is required) - the exercise has only been explained, demonstrated or shown during separate familiarization sessions |
| **ITEM 5** | Diagnostic Tools | Assessment | 2 points: results of ≥2 diagnostic tools (kinematics, kinetics, electromyography) are presented   - must be clearly presented in the text body or as table or in a figure   1 point: results of 1 diagnostic tool (kinematics, kinetics, electromyography) are provided   - must be clearly presented in the text body or as table or in a figure   0 points: no diagnostic tool was applied or associated data are not reported   - only stating that a method was used without presenting any related data |
|  | Progression & individualisation of programme variables | Intervention | 2 points: exercise intensity and/or volume progress & interindividual differences are assessed   - must be explicitely mentioned in the method section or in a table with specific information about individuality (methods and/or results) - progression of exercise intensity (synonymous with peak moment/force) or/and (in ambiguous cases) exercise volume (synonymous with repetitions, but more preferable with total impulse and/or time under tension)   1 point: progression of exercise intensity and/or volume   - must be explicitly mentioned in the method section or in a table - all participants perform the same regimen WITHOUT any reference to individual differences - Contradicting the intended purpose of high intensity NHE training, individual variations (additional weights or higher initial movement speed) at relatively high repetition numbers (>6)   0 points: no individual progression or not reported   - a progression that stronger athletes should perform more repetitions per set than beginners (e.g., FIFA 11+) - a short reference to an existing study/methodology without any specific details about progression |
| **ITEM 6** | Feedback of target movement speed | Assessment | 2 points: angle-time information are provided in real time to the participants by a monitor   - must be clearly visible in a figure, picture or sketch or/and (in ambiguous cases) must be explicitly mentioned in the methods - it is important to present continuous information during the exercise where the participant's body should be situated at every single point in time   1 point: average cadence provided e.g., by a metronome   - must be explicitly mentioned in the methods - the use of a metronome is recommended, however orally given cadence is tolerated despite its potential inaccuracy   0 points: no feedback or not reported   - a short reference to an existing study/methodology without any appropriate details about feedback of target movement speed (e.g., slowest possible speed) |
|  | Feedback to execution quality | Intervention | 2 points: visually (real time feedback on a monitor) and audibly (e.g., by a coach or physiotherapist)   - must be explicitly mentioned in the methods (in the text or in an image/sketch) which kind of and how feedback was given (details about tools, specific aspects and/or purpose of feedback)   1 point: visually (real time feedback on a monitor) or audibly (e.g., by a coach or physiotherapist)   - must be explicitly mentioned in the methods which kind of feedback was given - it is recommended that the participant-to-instructor ratio does not exceed 2:1 - if special emphasis is directed to 'individual training' (participant-to-instructor ratio does not exceed 2:1) 1 point is awarded   0 points: no feedback or not reported   - a short statement that NHE training was supervised without any details about extent or content of feedback - a short reference to an existing study/methodology without any specific details about feedback to execution quality |
| **ITEM 7** | Consequences of impaired technique | Assessment | 1 point: defined consequences (e.g., repeated or excluded from analysis)   - must be explicitely mentioned in the method section - optimally a definition of parameters is provided how impaired technique is detected/characterized (e.g., hip flexion of more than 30°, lower back arch, 20% deviation from target movement speed)   0 points: unclear consequences or not reported   - a short reference to an existing study/methodology without any appropriate details about consequences of impaired technique |
|  | Inter-set rest | Intervention | 1 point: adequate rest of ≥3 min (inter-repetition rest of ~6 s between eccentric NHEs)   - must be explicitely mentioned in the method section or in a table - if an inter-repetition rest of >6 s was guaranteed, an inter-set rest period of ≥2 min is awarded with 1 point - it is strongly recommended to specify detailed information about inter-set and inter-repetition rest periods   0 points: inadequate rest or not reported   - a short reference to an existing study/methodology without any specific details about set rest |
| **ITEM 8** | Presentation of NHE performance variables | Assessment | 2 points: moment-angle or angle-time information   - must be presented and/or illustrated in the results section - typically in form of an informative figure - it is recommended to present continuous graphs, however e.g., data of at least three averaged ROM epochs are acceptable as well   1 point: information about time under tension or range of motion   - must be presented and/or illustrated in the results section (typically in form of a table)   0 points: no information available |
|  | Compliance | Intervention | 2 points: participants performed ≥85% of NHEs repetitions   - the compliance to the intended NHE intervention volume of the analyzed participants must be explicitely mentioned - it is required to report compliance to sessions, sets and/or repetitions specific to NHE   1 point: participants performed 66-85% of NHEs repetitions   - the compliance to the intended NHE intervention volume of the analyzed participants must be explicitely mentioned - it is recommended to report compliance to sessions, sets and/or repetitions   0 points: participants performed <66% of NHEs repetitions or not reported   - in case of complex interventions where NHE is only one selective exercise, the detailed NHE compliance should be stated; if only a whole intervention compliance is reported, 0 points are awarded |
|  | General guidlines | Assessment and Intervention | - a short reference to an existing study/methodology without any appropriate details is rated as the reference - if a reference is made to an existing study, detailed descriptions should be added, otherwise 0 points are awarded - in case of doubt about the awarding of 2, 1 or 0 points, still the worse grading should be applied - the summated ANHEQ scores are allocated to grades according to American college grading system as: 12/13 points „excellent“; 11/10 points „very good“; 9/8 points „good“; 7/6 points „average“; 5/4 points „below average“; 3/2 points „poor“; 1/0 points „failure“) |
